# Anxiety and Depression in Patients with Obstructive Sleep Apnoea before and after Continuous Positive Airway Pressure: The ADIPOSA Study

**DOI:** 10.3390/jcm8122099

**Published:** 2019-12-01

**Authors:** Almudena Carneiro-Barrera, Francisco J. Amaro-Gahete, Germán Sáez-Roca, Carlos Martín-Carrasco, Jonatan R. Ruiz, Gualberto Buela-Casal

**Affiliations:** 1 Sleep and Health Promotion Laboratory, Mind, Brain and Behaviour Research Centre (CIMCYC), University of Granada, 18011 Granada, Spain; gbuela@ugr.es; 2 EFFECTS-262 Research group, Department of Medical Physiology, School of Medicine, University of Granada, 18071 Granada, Spain; amarof@ugr.es; 3 PROmoting FITness and Health through physical activity research group (PROFITH), Sport and Health University Research Institute (iMUDS), Department of Physical Education and Sports, Faculty of Sport Sciences, University of Granada, 18071 Granada, Spain; ruizj@ugr.es; 4 Unidad de Trastornos Respiratorios del Sueño, Servicio de Neumología, “Virgen de las Nieves” University Hospital, 18014 Granada, Spain; gsaezroca@gmail.com (G.S.-R.); cmartincarrasco@gmail.com (C.M.-C.)

**Keywords:** obstructive sleep apnoea, depression, anxiety, continuous positive airway pressure, OSA, CPAP, dysthymia, euthymia, negative affect, positive affect

## Abstract

The prevalence and treatment response of depression and anxiety symptoms in obstructive sleep apnoea (OSA), although widely addressed in research and clinical settings, still remain unclear due to overlapping symptoms. The ADIPOSA study sought to elucidate the presence of non-overlapping symptoms of depression and anxiety in patients with moderate to severe OSA before and after continuous positive airway pressure (CPAP) treatment. Forty-eight adults aged 18–80 (68.75% men) with moderate to severe OSA were enrolled in this twelve-week longitudinal single-arm trial and completed a full-night ambulatory sleep diagnostic test and an assessment of cognitive-affective depression and anxiety symptoms using the Beck-Depression Inventory-Fast Screen (BDI-FS), the State-Trait Depression Inventory (IDER) and the State-Trait Anxiety Inventory (STAI). We found no cognitive-affective depression or anxiety symptoms of clinical relevance at baseline. The amelioration of depression and anxiety symptoms after CPAP use was only statistically significant when considering anxiety-trait (*p* < 0.01; *d* = 0.296) and euthymia (*p* < 0.05; *d* = 0.402), the distinctive component of depression. Although dysthymia or high negative affect remained unchanged, CPAP may be effective at reducing the lack of positive affect, a well-established health-protective factor. However, not until depression and anxiety disorders related to OSA are accurately measured in clinical and research settings will it be possible to obtain robust conclusions on the occurrence and amelioration of these symptoms after treatment.

## 1. Introduction

Obstructive sleep apnoea (OSA) is the most common sleep-disordered breathing in the overall population, affecting up to 38% of adults, with higher prevalence in men, the elderly and in those who are overweight/obese [1]. Characterized by repeated total (apnoea) or partial (hypopnoea) upper airway collapse during sleep, OSA has globally become a major health concern due not only to its increasing prevalence—attributed to the current epidemic of obesity [2]—but also to its numerous and severe health-related consequences [3]. The intermittent pharyngeal obstructions during sleep lead to hypoxic and hypercapnic episodes, sleep disruption and increased/abnormal sympathetic activity [4]. These immediate consequences, in turn, may yield a vast number of physical, neurocognitive and neurobehavioral disturbances such as metabolic alterations [5,6], type II diabetes mellitus [7,8], life-threating cardiovascular diseases [9], impaired daytime functioning including memory and attention/concentration deficits [8,10], and mood and anxiety disorders [11]. Therefore, OSA is associated with higher morbidity and mortality [12], also including higher incidence of motor vehicle and workplace accidents mainly caused by excessive daytime sleepiness [13,14].

OSA is diagnosed when (1) there is a presence of five or more obstructive respiratory events per hour of sleep or per hour of recording time determined by polysomnography or out-of-centre sleep study, accompanied by one or more of the following symptoms: (a) sleepiness, fatigue, non-restorative sleep, or insomnia symptoms; (b) gasping or choking during sleep; (c) snoring and breathing interruptions reported by the bed partner; or (d) hypertension, mood disorder, cognitive dysfunction, stroke, coronary artery disease, congestive heart failure, atrial fibrillation, or type II diabetes mellitus; or (2) the polysomnography or out-of-centre sleep study shows fifteen or more obstructive respiratory events per hour of sleep or per hour of recording time, respectively, independent of the presence of other symptoms, complaints or comorbidities [15].

It has been well evidenced that depression, the most common affective disorder found in OSA [16,17,18], is also related to the occurrence and worsening of severe medical conditions such as metabolic and cardiovascular diseases [19], besides being a major contributing factor to non-compliance with OSA treatment [20]. According to a recent study on the prevalence of OSA in psychiatric disease [21], moderate to severe OSA can be found in 72.48% of patients with affective and psychotic disorders, so the consideration of mood disturbances should play an important role in both the evaluation and treatment of this condition. Recent epidemiological studies have also indicated that anxiety may be found in 53.9% of patients with OSA, associated with higher body mass index (BMI) and severity of OSA [11], and once again with poor compliance to OSA therapy [20].

Continuous positive airway pressure (CPAP) is the gold standard treatment for OSA [22,23], which consists of a mechanical device that prevents upper airway collapse during sleep. Numerous studies have shown that CPAP is an effective treatment in improving OSA primary outcomes such as the number of episodes of apnoea and hypopnoea per hour of sleep (i.e., apnoea-hypopnoea index, AHI) and excessive daytime sleepiness, reducing therefore the majority of symptoms and consequences of this condition [24,25,26,27]. However, the effectiveness of CPAP in addressing OSA major risk factors, i.e., obesity, still remains unclear. Some studies found weight loss after a twelve-week CPAP therapy [28], while others highlighted that CPAP was not related to BMI change or induced body weight gain [29,30], although the latter may be due to increased lean mass as opposed to fat mass [31]. The latest American Academy of Sleep Medicine (AASM) guidelines therefore strongly recommend weight loss and lifestyle intervention combined with CPAP [22,32], which appears to be an effective treatment for moderate to severe OSA (i.e., AHI ≥ 15 events/h [22]) and is the focus of current interdisciplinary clinical and medical research [3,33].

Similarly, there have been inconsistent findings regarding CPAP effects on OSA associated depression and anxiety symptoms [34]. While some clinical research found that a twelve-week CPAP use significantly reduced depression symptoms in patients with moderate to severe OSA [35,36,37], other studies indicated no significant changes in these psychological disturbances after CPAP treatment [38,39,40]. These controversial results may be explained by both (a) cofounder variables in the association between anxiety-depression and OSA, and (b) commonly used anxiety and depression scales.

OSA and depression are both independently related to obesity, increasing age, and adverse lifestyles, sharing common symptomatology such as daytime sleepiness, insomnia, fatigue and poor quality of life [34]. Consequently, improvement of OSA main outcome (i.e., AHI) after CPAP use may not necessarily result in a reduction in depressive symptoms due to potential remaining factors such as obesity and unhealthy habits [29]. In turn, anxiety and depression scales commonly used in order to measure baseline and posttreatment mood disturbances in patients with OSA (e.g., Hamilton Depression Rating Scale [41], Beck Depression Inventory [42], and Beck Anxiety Inventory [43]) usually include somatic symptoms found in and caused by OSA such as cardiovascular palpitations, headaches, gastrointestinal and genitourinary disturbances, loss of energy and libido, and fatigue, such that high scores of these symptoms at baseline may not necessarily signify presence of depression in patients with OSA. Therefore, further evidence including anxiety and depression measures through valid scales adjusted to medical patients—solely including non-overlapping symptoms such as cognitive-affective symptomatology—are needed not only to establish the presence of anxiety and depression disturbances in patients with OSA but also to clarify the effectiveness of CPAP therapy in reducing these specific and adverse outcomes.

The ADIPOSA study was aimed at establishing the presence of non-overlapping symptoms (i.e., cognitive-affective symptoms) of depression and anxiety in patients with moderate to severe OSA before and after a twelve-week CPAP therapy. Particularly, we hypothesised that there would be no depression nor anxiety symptomatology of clinical relevance in patients with OSA when only considering cognitive-affective symptoms. In turn, we also expected that, although a twelve-week CPAP treatment would be effective at reducing OSA primary outcome (i.e., AHI), there would be no relevant changes on cognitive-affective symptoms of depression and anxiety from baseline to posttreatment.

## 2. Experimental Section

### 2.1. Study Protocol and Participant’s Enrolment

The Anxiety and Depression in Patients with Obstructive Sleep Apnoea Before and After Continuous Positive Airway Pressure (ADIPOSA) study was a twelve-week longitudinal single-arm trial of CPAP treatment. The study protocol was approved by the Human Research Ethics Committees of Junta de Andalucía (0307-N-19) and registered online in a specific clinical trial database (i.e., Clinicaltrial.gov. ID: NCT03334357). The recruitment of participants was performed from April to June 2019, with patients being enrolled from the “Virgen de las Nieves” University Hospital (Granada, Spain). Eligible participants were adults aged 18–80 years, with a clinical suspicion of OSA and a BMI greater than 25 kg/m^2^. The study exclusion criteria included current or recent CPAP treatment (i.e., during the last year) and presence of (i) other primary sleep disorders (e.g., shift work disorder, idiopathic hypersomnia and/or narcolepsy), (ii) a previously diagnosed mental disorder hindering participant’s ability to perform study assessments, and/or (iii) any other severe organic diseases excluding those comorbid to OSA. Participants regularly using neuroleptic, antidepressant, sedative or hypnotic drugs, or any other medication that may cause sleep disturbances or increased daytime sleepiness were also excluded from the study.

### 2.2. Procedures

Upon meeting the inclusion criteria, informed consent signatures were obtained from potential participants with OSA willing to be enrolled. Participants were medically examined, and sociodemographic data and general health outcomes (e.g., sex, birth date, medical history, alcohol consumption and smoking history among others) were collected. Height and weight measurements were completed with an electronic scale (SECA, model 799, Electronic Column Scale, Hamburg, Germany), and were used to determine BMI by dividing weight (kg) by the square of height (m). An overnight sleep study and subjective measurements of daytime sleepiness, depression and anxiety symptoms were performed both at baseline and after a twelve-week CPAP treatment for all patients.

#### 2.2.1. Overnight Sleep Study

A full-night ambulatory cardiorespiratory polygraphy (SOMNOScreen™ plus RM-Tele, SOMNOmedics, GmbH, Randersacker, Germany)—a widely validated and accepted OSA diagnostic tool [22,44,45]—was conducted on each participant in order to determine the number of apnoea-hypopnoea episodes per hour of valid total recording time (i.e., AHI). Recordings were analysed by trained sleep physicians using a specific software (i.e., DOMINO v2.7, SOMNOmedics, GmbH, Randersacker, Germany) following the AASM Manual for the Scoring of Sleep and Associated Events [46]. In accordance with the latter, AHI was defined as the number of apnoea (90% or greater airflow drop lasting 10 s or longer) and hypopnoeas (30% or greater airflow drop lasting 10 s or longer and associated ≥ 3% oxygen desaturation) episodes per hour of valid total recording time [46]. Consequently, patients were diagnosed with moderate (15 events/h ≤ AHI < 30 events/h) or severe OSA (AHI ≥ 30 events/h).

#### 2.2.2. Daytime Sleepiness, Depression and Anxiety

Subjective daytime sleepiness was assessed by the Epworth Sleepiness Scale [47,48], a widely used and reliable 8 item Likert-based scale that provides an accurate score of propensity for dozing during several daily activities from none to high (0–3). Hypersomnolence is considered when a total score greater than 10 is attained.

General and state-trait depression symptoms were assessed using the Beck-Depression Inventory-Fast Screen (BDI-FS) [49,50] and the State-Trait Depression Inventory (IDER) [51], respectively. Both instruments are reliable depression screening tools for medical patients which solely include those symptoms of depression that do not overlap with somatic disturbances caused by other physical conditions, i.e., cognitive-affective symptoms of depression. BDI-FS consists of a 7 item Likert-based scale related to sadness, pessimism, past failure, loss of pleasure, self-dislike, self-criticalness, and suicidal thoughts. Previous studies have emphasised the excellent psychometric properties and advantages of this scale for the screening of depression symptoms not only in patients with OSA [52] but also in patients with other medical conditions [53]. Similarly, IDER evaluates non-overlapping symptoms of depression but, in contrast to BDI-FS, also includes a differentiation between state and trait depression symptoms (IDER-state and IDER-trait), as well as dysthymia (high negative affect) and euthymia (lack of positive affect) components. This instrument is composed of 10 items for each subscale of state and trait depression symptoms (i.e., a total of 20 items), with answers ranging from “not at all” to “very much so” (0–4), and from “almost never” to “almost always” (0–4), respectively. Both direct scores and converted percentiles of each participant were considered for the interpretation of results.

The intensity and frequency of displaying anxiety symptoms were determined by the State-Trait Anxiety Inventory (STAI) [54,55]. This questionnaire includes a total of 40 items, 20 for the State Anxiety subscale (STAI-State) and 20 for the Trait Anxiety subscale (STAI-Trait). STAI-State evaluates the current state of anxiety by obtaining answers ranging from “not at all” to “very much so” (0–3) to several items (i.e., worry, apprehension, nervousness, tension and autonomic nervous system activation/arousal) depending on how they feel “at this moment”. STAI-Trait assesses stable aspects of “anxiety proneness” or the frequency of feeling anxious (i.e., general states of confidence, calmness, or security). STAI-Trait answers range from “almost never” to “almost always” (0–3). Both direct scores and converted percentiles of each participant were also considered for the interpretation of results.

### 2.3. CPAP Usage

A CPAP device (Oximesa oxygen company, Madrid, Spain), as well as a tube with an embedded heating circuit and a humidifier, were provided to each participant after the baseline assessment as medically recommended. In addition, an appropriate mask was selected according to participant’s facial dimensions. A qualified sleep physician instructed participants on how to use the CPAP automatic mode during the first week in order to manually program the CPAP pressure during the rest of the intervention period (CPAP pressure was usually the 95th centile). CPAP use and pressure were defined as the average of (i) daily usage of CPAP (h/per night), and (ii) daily pressure of CPAP (mmHg), respectively, in accordance with clinical guidelines [56].

### 2.4. Sample Size Calculation

The sample size calculation was performed expecting an effect size of −0.5 on BDI-FS score after CPAP intervention with a variability (SD) of 1 (based on a previous study [52] and on our own previous observations), using the formula $n=\frac{2{\left(Z_{\alpha }\;+\;Z_{1-\beta }\right)}^{2}\sigma^{2}}{\Delta^{2}}$. A sample size of ≈36 participants was therefore expected to provide a statistical power of 85% (*Z*_(1−*β*)_ = 1.28) considering a type I error of 0.05 (*Z_α_* = 1.96) [57]. Assuming a maximum loss of 20% at follow-up, we decided to recruit a minimum of 45 participants.

### 2.5. Statistical Analysis

Descriptive parameters are expressed as the mean (SD) for continuous variables and number of patients (%) for categorical variables. Visual check histograms, quantile-quantile (Q-Q) plots and the Shapiro–Wilk test were used to confirm the normality of our data. Unpaired *t*-student tests were performed to study potential differences between sexes at baseline assessment. As no interactions by sex were noted, the appropriateness of fitting models included both men and women.

Paired *t*-student tests were conducted to study changes in AHI, Epworth sleepiness score, BDI-FS score, STAI-State and STAI-Trait scores, and IDER-State and IDER-Trait scores after CPAP therapy. Standardized effect size coefficients (*d*) were computed as the mean difference between mean baseline and mean post-intervention values, divided by mean baseline standard deviation $d=c\left(df\right)\cdot \left[\left({\bar{X}}_{pre,E}-{\bar{X}}_{pos,E}\right)/{\bar{S}}_{pre,E}\right]$ where *c(df)* is a correction factor for small samples [58].

Similar analyses were separately performed (i) on overweight and obese patients, (ii) on patients with moderate and severe OSA, and (iii) on men and women. An analysis of covariance was additionally conducted to examine whether or not the above-mentioned changes persisted after controlling for sex, age, CPAP use and BMI.

Simple linear regression models were built to investigate (i) the association of the Epworth sleepiness score, BDI-FS score, STAI-State and STAI-Trait scores, and IDER-State and IDER-Trait scores with AHI, and (ii) the association of changes in the above-mentioned outcomes with changes in AHI. Multiple linear regression models were also performed to test these associations adjusting for sex (Model 1), age (Model 2), CPAP use (Model 3), and BMI (Model 4). Additionally, a simple linear regression was conducted in order to study the association of BDI-FS score, STAI-State and STAI-Trait scores, and IDER-State and IDER-Trait scores with Epworth sleepiness scores and BMI.

The Statistical Package for Social Sciences (SPSS, v. 22.0, IBM SPSS Statistics, IBM Corporation) was used to perform the analyses, and the GraphPad Prism 5 (GraphPad Software, San Diego, CA, USA) were used to build graphical plots. The level of significance was set at <0.05.

## 3. Results

Table 1 shows the baseline characteristics of the study participants. CPAP use ranged from 1.0 to 8.1 h. Differences by sex where only statistically significant when considering age of participants. No significant changes in BMI were observed after the twelve-week CPAP intervention (*p* > 0.5). Overall, no presence of cognitive-affective symptoms of depression and anxiety were observed in the study participants (see Table 1 percentiles).

**Figure 1:**
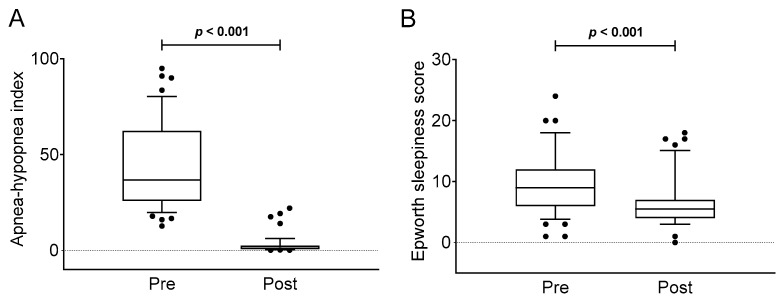
Apnoea-hypopnoea index (Panel **A**) and Epworth sleepiness score (Panel **B**) before and after the continuous positive airway pressure therapy. P value of Student’s paired t-test. The data are shown as the median ± interquartile range.

Figure 1 shows changes in AHI and sleepiness after CPAP treatment. Significant reductions in AHI (*p* < 0.001, *d* = 1.809; Figure 1A and Table 2) and Epworth sleepiness score (*p* < 0.001, *d* = 0.612; Figure 1B and Table 2) were observed.

**Figure 2:**
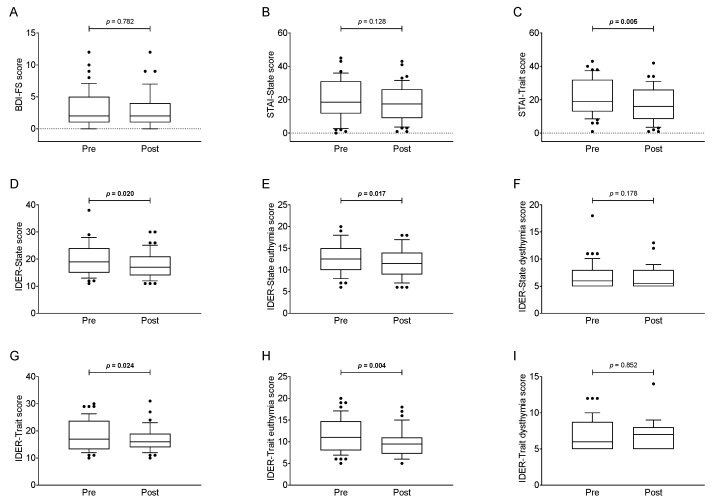
Beck Depression Inventory-FS (BDI-FS) score (Panel **A**), State and Trait Anxiety Inventory (STAI) scores (Panel **B**,**C**), and State-Trait Depression Inventory (IDER) scores (Panel **D**–**I**) before and after the continuous positive airway pressure therapy. *p* value of Student’s paired *t*-test. The data are shown as the median ± interquartile range.

Changes in general and state-trait depression symptoms, as well as in the intensity and frequency of displaying anxiety symptoms, can be seen in Figure 2. Significant reductions in STAI-Trait score (*p* = 0.005, *d* = 0.413; Figure 2C and Table 2), IDER-State (*p* = 0.020, *d* = 0.274; Figure 2D and Table 2) and IDER-Trait score (*p* = 0.024, *d* = 0.296; Figure 2G and Table 2) were observed, whereas no significant changes in BDI-FS (*p* = 0.782, *d* = 0.042; Figure 2A and Table 2) and STAI-State (*p* = 0.128, *d* = 0.181; Figure 2B and Table 2) scores were noted. Moreover, a significant decrease in IDER-State euthymia and IDER-Trait euthymia scores were found (*p* = 0.017, *d* = 0.328 and *p* = 0.004, *d* = 0.402, respectively; Figure 2E,H, and Table 2), while no significant changes were observed in IDER-State dysthymia and IDER-Trait dysthymia scores after the CPAP intervention (*p* = 0.178, *d* = 0.156 and *p* = 0.852, *d* = 0.028, respectively; Figure 2F,I, and Table 2). All above-mentioned findings persisted not only after dividing our study sample in overweight and obese patients (Supplementary Figures S1 and S2), but also after categorizing our cohort as patients with moderate vs. severe OSA (Supplementary Figures S3 and S4) and after separating men and women (Supplementary Figures S5 and S6). These results did not change after controlling for sex, age, CPAP use and BMI.

No association between Epworth sleepiness, BDI-FS, STAI-State, STAI-Trait, IDER-State, IDER-Trait, IDER-State euthymia, IDER-Trait euthymia, IDER-State dysthymia and IDER-Trait dysthymia scores, and AHI (Supplementary Figures S7 and S8; all *p* > 0.2) was detected at baseline, which remained non-significant adjusting for several confounders (all *p* > 0.1; Supplementary Table S1).

No significant associations were observed between changes in Epworth sleepiness, BDI-FS, STAI-State, STAI-Trait, IDER-State, or IDER-Trait, IDER-State euthymia, IDER-Trait euthymia, IDER-State dysthymia and IDER-Trait dysthymia scores and changes in AHI after CPAP therapy (all *p* > 0.15; Supplementary Figures S9 and S10), which persisted after adding sex, age, CPAP use and BMI as covariates (all *p* > 0.16; Supplementary Table S2).

No significant associations were found between BDI-FS, STAI-State, STAI-Trait, IDER-State, IDER-Trait, IDER-State euthymia, IDER-Trait euthymia, IDER-State dysthymia or IDER-Trait dysthymia scores and BMI (all *p* > 0.14; Supplementary Figures S10 and S11). Similarly, Epworth sleepiness score was not related to BDI-FS, STAI-State, STAI-Trait, IDER-State nor IDER-Trait (all *p* > 0.6; Supplementary Figures S11 and S12).

Additionally, a positive association was found between BMI and AHI (*p* = 0.008), while no significant association was observed between BMI and Epworth sleepiness score (*p* = 0.694) (Supplementary Figure S13).

## 4. Discussion

The ADIPOSA study sought to elucidate the presence of non-overlapping symptoms of depression and anxiety in patients with moderate to severe OSA before and after a twelve-week CPAP treatment. As we expected, although certain cognitive-affective symptoms of depression and anxiety were found at baseline, scores of these symptoms were not of clinical relevance. Furthermore, the amelioration of depression and anxiety symptomatology after a twelve-week CPAP therapy was only statistically significant when considering changes in anxiety-trait and both euthymia (i.e., lack of positive affect) trait and state outcomes. Our findings therefore may elucidate what has been indicated in previous research regarding a potential overestimation of depression and anxiety disturbances in OSA due to overlapping somatic symptoms found in both OSA and depression/anxiety, which are included in the scales commonly used for the assessment of these symptoms [59].

OSA and depression are both prevalent adverse medical and psychological conditions independently related to obesity and unhealthy lifestyles, sharing common diagnostic criteria such as daytime sleepiness, insomnia, fatigue and poor quality of life [34]. Therefore, there are inconsistent findings regarding the direct relationship between these conditions [34,59]. Our results indicated that, when solely assessing non-overlapping depression symptoms—i.e., cognitive-affective symptoms—patients with OSA did not display a depression level of clinical relevance since scores in BDI-FS and percentiles in IDER were under 4 and 75, respectively [49,51]. This finding is in accordance with previous epidemiological research emphasising that depression prevalence on OSA drastically varies from 68.33% to 7.59% depending on whether the depression questionnaire used included overlapping symptoms or not, respectively [59].

Furthermore, the great impact of the different assessment tools used for depression in OSA may also be the key factor explaining the controversy found in research on the effectiveness of CPAP treatment in reducing these mood disturbances [34]. According to our results, there were no general changes in depression symptoms measured with BDI-FS on patients with moderate to severe OSA after a twelve-week CPAP use. Nonetheless, we found statistically significant specific changes in both depression state and depression trait from baseline to posttreatment when measuring with IDER. Although these results may appear controversial at first glance, they expose an original and remarkable difference which has not previously been addressed in this field. While BDI-FS measures cognitive-affective depression symptoms based on negative affect exclusively, IDER assesses not only the high negative affect (i.e., dysthymia) found in depression but also the lack of positive affect (i.e., euthymia), a defining factor of this mood disorder [60]. Our results indicate changes in depression symptoms after CPAP use only when considering positive affect, i.e., patients with OSA tended to interpret situations more optimistically or feel more positive emotions such as joy, happiness and hope after CPAP use, although the intensity and frequency of feeling negative emotions remained unchanged. Consistent with previous research [60], low positive affect is the distinctive feature of depression whereas high negative affect is a component of both depression and anxiety, so an improvement in the tendency of feeling negative emotions would require reductions in both depression and anxiety symptoms.

This noteworthy result may be of relevance to clinical and research practice as, although negative affect has been associated to non-compliance to CPAP use [61], positive affect has generally been closely related to lower allostatic load profiles including neuroendocrine, inflammatory, cardiovascular and metabolic biomarkers such as levels of cortisol, C-reactive protein, systolic blood pressure, diastolic blood pressure, heart rate, total cholesterol, triglycerides, low-density lipids, high-density lipids, albumin, glucose, HbA1c, and waist circumference [62,63,64]. As alterations of these neuroendocrine, inflammatory, cardiovascular and metabolic markers, in turn, are causes and consequences of not only OSA but also other severe chronic conditions [65,66], addressing positive affect may play an important role in the assessment and treatment of these conditions.

Regarding potential differences on cognitive-affective depression symptoms by sex, BMI and OSA severity, there were no differences in the presence of these symptoms mediated by any of these variables. These results are in accordance with previous epidemiological studies where depression was not correlated with sex, BMI or OSA severity [11]. In contrast, other studies found higher prevalence rates of depression in women and in those with higher BMI and severe OSA [67]. As previously noted, these inconsistent findings may be explained by the cofounder variables between OSA and depression and, therefore, the scales frequently used in observational studies. Similarly, changes found in depression symptoms (i.e., euthymia) after CPAP therapy in our study were not related to sex, age, BMI, CPAP compliance, changes in AHI nor daytime sleepiness. A preceding study with similar results highlighted that changes in mood disturbances after CPAP use, rather than simply and directly related to reduced AHI or daytime sleepiness, are linked to an increased quality of life of these patients [68], which once again exposes the complex and still unclear relationship between OSA and depression.

The association between OSA and anxiety has also been consistently established throughout a large number of epidemiological research [11,69]. In our study, after a twelve-week CPAP treatment, patients with OSA showed significant reductions in the anxiety-trait outcome, which has also been found in previous research [35]. Thus, patients with moderate to severe OSA, although displaying transitory symptoms of nervousness, tension and/or apprehension, tended to perceive situations as less threatening or alarming than before CPAP use. These results may be mainly explained by the general increased quality of life of patients with OSA after CPAP treatment [27].

To our knowledge, this is the first study on the presence and changes after CPAP therapy of cognitive-affective depression and anxiety symptoms in patients with OSA including not only the measurement of dysthymia but also the distinctive component of depression, i.e., euthymia. Although the prevalence and treatment response of these symptoms in OSA have been widely addressed in previous epidemiological and original research [16,17,18,34,35,36,37,38,39,40], the use of mood disturbances scales including overlapping symptoms, as well as the non-consideration of euthymia as the distinctive feature of depression, have potentially led to inconsistencies and unclear results in this field. Our results therefore have robust clinical and research implications, further supporting the potential overestimation of depression and anxiety prevalence in patients with OSA found throughout the large body of literature in this field [59]. Due to mood disturbances such as depression being a specific symptom included in the diagnostic criteria of OSA [15], only cognitive-affective components of depression should be measured when establishing the diagnosis of this sleep-disordered breathing, using scales adjusted to medical patients in order to perform an accurate OSA diagnosis which in turn will determine the posterior treatment. Furthermore, this study adds an interesting and previously disregarded factor to the research on depression and OSA association, i.e., the distinction between dysthymia and euthymia when assessing and addressing mood disturbances in patients with this condition and others. As emphasised by the World Health Organisation, depression is an important leading cause of disease burden worldwide highly comorbid to the majority of chronic physical conditions [70]. Therefore, our main findings could be cautiously generalised to the assessment and treatment of depression in other chronic diseases such as type II diabetes mellitus, metabolic syndrome, asthma, angina and arthritis.

Nevertheless, our findings should be interpreted with caution as these are limited to the design and methodology followed in our study. The main limitation was the lack of a comparison (i.e., control) group which could have fully allowed us to determine the cause-effect relationship between treatment and results. The unequal number of men and women included in our sample, although consistent with the well-established higher prevalence of OSA in men [1], was another limitation. A balanced sample by sex could have been a determining factor in order to analyse potential differences between men and women previously found in this area. However, we did not find any interaction by sex in our study, which indicates that the effect of the intervention was similar in men and women. Another limitation of our study is that, as the sample included did not displayed depression nor anxiety symptoms of clinical relevance at baseline, potential benefits of CPAP therapy for the amelioration of these symptoms may not have been properly assessed. The measurement of daytime sleepiness, depression and anxiety symptoms by subjective measures, though widely used and reliable scales [47,48,49,50,51,52,53,54,55], may have also been a limitation as they are not as accurate as objective measures. Similarly, accurate measures of other interesting outcomes such as quality of life, exercise practice and nutritional habits, not included in our study, could have enabled us to analyse potential interactions of these variables on the occurrence and adjustment of depression and anxiety symptoms in OSA.

Future well-designed observational and experimental studies are therefore required to further support these evidences. In order to elucidate both the prevalence of mood disturbances and the effectiveness of OSA treatments for these symptoms, epidemiological studies and randomised controlled trials with larger and balanced samples should only include accurate measurements of non-overlapping cognitive-affective depression and anxiety symptoms adjusted to medical patients. Depression measurements, specifically, should not only include dysthymia or negative affect component but also lack of positive affect or euthymia, the idiosyncratic component of depression [60]. Finally, as quality of life and general well-being has been closely related to depression [71]—with sleep quality as a feasible mediating factor [72]—, further studies should also include appropriate measurements of these variables and others such as exercise practice and nutritional habits.

## 5. Conclusions

The ADIPOSA study further highlights the essential need to use precise and accurate depression and anxiety scales adjusted to medical patients not only for assessment purposes but also for the treatment of these symptoms in patients with OSA. According to our findings, the prevalence of mood disturbances and anxiety in patients with this condition may have been overestimated throughout the large body of evidence, as we did not find a clinically relevant occurrence of non-overlapping depression and anxiety symptoms. The distinction of both dysthymia and euthymia components of depression may also be crucial when assessing treatment effects on this mood disturbance. In this regard, we found that a twelve-week CPAP therapy was specifically effective at improving the lack of positive affect, a potential health-protective factor of relevance. However, not until depression and anxiety disorders related to chronic diseases are accurately measured in clinical and research settings will it be possible to obtain robust conclusions on occurrence and amelioration after medical and/or psychological treatment of these disturbances.

## Figures and Tables

**Table 1:** Descriptive characteristic of participants.

	*N*	All		*N*	Men		*N*	Women	
Age (years)	48	54.5	(13.1)	33	51.0	(12.0)	15	62.0	(12.6) *
Body mass index (kg/m^2^)	48	32.8	(6.8)	33	33.5	(7.3)	15	31.2	(5.4)
Smokers (*n*, %)	48	8	(16.7)	33	7	(21.2)	15	1	(6.7)
Alcohol consumers (*n*, %)	48	5	(10.4)	33	5	(15.2)	15	0	(0.0)
OSA severity classification									
Moderate OSA (*n*, %)	48	13	(27.1)	33	8	(24.2)	15	5	(33.3)
Severe OSA (*n*, %)	48	35	(72.9)	33	25	(75.8)	15	10	(66.7)
Apnoea-hypopnoea index (*n*)	48	44.2	(22.5)	33	47.0	(23.3)	15	38.0	(19.9)
CPAP use (h)	48	5.7	(1.8)	33	5.9	(1.6)	15	5.3	(2.2)
CPAP pressure (mmHg)	48	9.7	(1.8)	33	9.5	(2.0)	15	9.9	(1.2)
Epworth questionnaire									
Epworth sleepiness score	47	9.85	(5.27)	32	9.94	(5.16)	15	9.67	(5.69)
BDI-FS questionnaire									
BDI-FS score	48	2.92	(3.05)	33	2.82	(2.90)	15	3.13	(3.44)
Sadness	48	0.25	(0.48)	33	0.24	(0.44)	15	0.27	(0.59)
Pessimism	48	0.50	(0.74)	33	0.52	(0.76)	15	0.47	(0.74)
Past failure	48	0.29	(0.65)	33	0.27	(0.52)	15	0.33	(0.9)
Loss of pleasure	48	0.54	(0.74)	33	0.55	(0.75)	15	0.53	(0.74)
Self-dislike	48	0.48	(0.83)	33	0.45	(0.75)	15	0.53	(0.99)
Self-criticalness	48	0.77	(0.72)	33	0.70	(0.81)	15	0.93	(0.46)
Suicidal thoughts	48	0.08	(0.28)	33	0.09	(0.29)	15	0.07	(0.26)
STAI questionnaire									
STAI-State score	46	20.11	(12.57)	31	18.03	(12.82)	15	24.40	(11.24)
STAI-State score percentile ^a^	46	57.50	(33.90)	31	53.26	(37.24)	15	66.27	(24.49)
STAI-Trait score	45	21.60	(11.06)	30	20.97	(11.81)	15	22.87	(9.64)
STAI-Trait score percentile ^a^	45	51.62	(32.07)	30	53.50	(34.37)	15	47.87	(27.61)
IDER questionnaire									
IDER-State score	48	19.56	(5.67)	33	18.82	(5.86)	15	21.20	(5.03)
IDER-State score percentile ^a^	48	61.37	(26.17)	33	58.52	(28.28)	15	67.67	(20.25)
IDER-State euthymia score	48	12.71	(3.51)	33	12.21	(3.60)	15	13.80	(3.14)
IDER-State dysthymia score	48	6.85	(2.59)	33	6.61	(2.67)	15	7.40	(2.38)
IDER-Trait score	48	18.42	(5.62)	33	18.06	(5.33)	15	19.20	(6.35)
IDER-Trait score percentile ^a^	48	50.21	(32.09)	33	50.88	(32.25)	15	48.73	(32.82)
IDER-Trait euthymia score	48	11.46	(3.99)	33	11.27	(3.83)	15	11.87	(4.44)
IDER-Trait dysthymia score	48	6.96	(2.13)	33	6.79	(1.98)	15	7.33	(2.47)

Data are shown as the mean (standard deviation). * Significant differences between sexes were obtained from unpaired *t*-student tests (*p* < 0.05). ^a^ Score percentile variables refer to the mean and standard deviation of the percentiles for STAI-State, STAI-Trait, IDER-State, and IDER-Trait. Abbreviations: OSA, obstructive sleep apnoea; CPAP, continuous positive airway pressure; BDI-FS, Beck Depression Inventory-Fast Screen; STAI, State and Trait Anxiety Inventory; IDER, State-Trait Depression Inventory.

**Table 2:** Apnoea-hypopnoea index, Epworth sleepiness score, BDI-FS score, STAI-State and STAI-Trait scores, and IDER-State and IDER-Trait scores before and after the CPAP intervention.

	All (*n* = 48)			Men (*n* = 33)			Women (*n* = 15)		
	Pre Mean (SD)	Post Mean (SD)	d (95%CI)	Pre Mean (SD)	Post Mean (SD)	d (95%CI)	Pre Mean (SD)	Post Mean (SD)	d (95%CI)
Apnoea-hypopnoea index (*n*)	44.17 (22.51)	2.78 (4.90)	1.809 (1.262 to 2.344)	46.99 (23.35)	2.49 (4.78)	1.875 (1.319 to 2.420)	37.95 (19.89)	3.41 (5.27)	1.708 (1.175 to 2.230)
Epworth sleepiness score	9.85 (5.27)	6.57 (4.14)	0.612 (0.190 to 1.027)	9.94 (5.16)	6.28 (4.46)	0.699 (0.272 to 1.120)	9.67 (5.69)	7.20 (3.41)	0.427 (0.016 to 0.834)
BDI-FS score	2.92 (3.05)	2.79 (2.82)	0.042 (−0.358 to 0.442)	2.82 (2.90)	2.67 (3.12)	0.051 (−0.349 to 0.451)	3.13 (3.44)	3.07 (2.09)	0.017 (−0.383 to 0.417)
STAI-State score	20.11 (12.57)	17.80 (10.74)	0.181 (−0.222 to 0.582)	18.03 (12.82)	15.29 (9.98)	0.21 (−0.193 to 0.611)	24.40 (11.24)	23.00 (10.71)	0.123 (−0.278 to 0.523)
STAI-Trait score	21.60 (11.06)	16.96 (10.26)	0.413 (0.002 to 0.820)	20.97 (11.81)	14.23 (9.07)	0.562 (0.143 to 0.975)	22.87 (9.64)	22.40 (10.62)	0.048 (−0.352 to 0.448)
IDER-State score	19.56 (5.67)	17.98 (4.86)	0.274 (−0.131 to 0.676)	18.82 (5.86)	17.15 (4.79)	0.281 (−0.125 to 0.684)	21.20 (5.03)	19.80 (4.66)	0.274 (−0.131 to 0.676)
IDER-State euthymia score	12.71 (3.51)	11.54 (3.34)	0.328 (−0.079 to 0.732)	12.21 (3.60)	10.88 (3.32)	0.364 (−0.045 to 0.769)	13.80 (3.14)	13.00 (2.98)	0.250 (−0.155 to 0.652)
IDER-State dysthymia score	6.85 (2.59)	6.44 (1.91)	0.156 (−0.246 to 0.556)	6.61 (2.67)	6.27 (1.79)	0.125 (−0.276 to 0.525)	7.40 (2.38)	6.80 (2.18)	0.248 (−0.156 to 0.650)
IDER-Trait score	18.42 (5.62)	16.73 (4.23)	0.296 (−0.110 to 0.699)	18.06 (5.33)	16.55 (4.79)	0.279 (−0.126 to 0.682)	19.20 (6.35)	17.13 (2.72)	0.321 (−0.086 to 0.725)
IDER-Trait euthymia score	11.46 (3.99)	9.83 (3.09)	0.402 (−0.008 to 0.808)	11.27 (3.83)	9.76 (3.44)	0.388 (−0.022 to 0.794)	11.87 (4.44)	10.00 (2.24)	0.415 (0.004 to 0.822)
IDER-Trait dysthymia score	6.96 (2.13)	6.90 (1.81)	0.028 (−0.372 to 0.428)	6.79 (1.98)	6.79 (1.97)	0.001 (−0.399 to 0.401)	7.33 (2.47)	7.13 (1.46)	0.080 (−0.321 to 0.480)

Data are shown as the mean (standard deviation). Abbreviations: BDI-FS, Beck Depression Inventory-Fast Screen; STAI, State and Trait Anxiety Inventory; IDER, State-Trait Depression Inventory; CPAP, continuous positive airway pressure.

## Supplementary Materials

The following are available online at https://www.mdpi.com/2077-0383/8/12/2099/s1, Figure S1: Apnoea-hypopnoea index, Epworth sleepiness score, Beck Depression Inventory-FS (BDI-FS) score, and State and Trait Anxiety Inventory (STAI) scores before and after the continuous positive airway pressure therapy comparing patients with obstructive sleep apnoea having a body mass index (BMI) <30 kg/m^2^ vs. ≥30 kg/m^2^, Figure S2: State-Trait Depression Inventory (IDER) scores, including euthymia and dysthymia specific values before and after the continuous positive airway pressure therapy comparing patients with obstructive sleep apnoea having a body mass index (BMI) <30 kg/m^2^ vs. ≥30 kg/m^2^, Figure S3: Apnoea-hypopnoea index, Epworth sleepiness score, Beck Depression Inventory-FS (BDI-FS) score, and State and Trait Anxiety Inventory (STAI) scores before and after the continuous positive airway pressure therapy comparing patients with moderate (AHI < 30) vs. severe (AHI ≥ 30) obstructive sleep apnoea (OSA), Figure S4: State-Trait Depression Inventory (IDER) scores, including euthymia and dysthymia specific values before and after the continuous positive airway pressure therapy comparing patients with moderate (AHI < 30) vs. severe (AHI ≥ 30) obstructive sleep apnoea (OSA), Figure S5: Apnoea-hypopnoea index, Epworth sleepiness score, Beck Depression Inventory-FS (BDI-FS) score, and State and Trait Anxiety Inventory (STAI) scores before and after the continuous positive airway pressure therapy men vs. women, Figure S6: State-Trait Depression Inventory (IDER) scores, including euthymia and dysthymia specific values before and after the continuous positive airway pressure therapy men vs. women, Figure S7: Association of the Epworth sleepiness score, the Beck Depression Inventory-FS (BDI-FS) score, and the State and Trait Anxiety Inventory (STAI) scores with the apnoea-hypopnoea index, Figure S8: Association of the State-Trait Depression Inventory (IDER) scores including euthymia and dysthymia specific values with the apnoea-hypopnoea index, Figure S9: Association of changes in the Epworth sleepiness score, the Beck Depression Inventory-FS (BDI-FS) score, and the State and Trait Anxiety Inventory (STAI) scores with changes in the apnoea-hypopnoea index after a post-continuous positive airway pressure therapy, Figure S10: Association of changes in the State-Trait Depression Inventory (IDER) scores including euthymia and dysthymia specific values with changes in the apnoea-hypopnoea index after a post-continuous positive airway pressure therapy, Figure S11: Association of the Beck Depression Inventory-FS (BDI-FS) score, and the State and Trait Anxiety Inventory (STAI) scores with the Epworth sleepiness score and the body mass index, Figure S12: Association of the State-Trait Depression Inventory (IDER) scores including euthymia and dysthymia specific values with the Epworth sleepiness score and the body mass index, Figure S13: Association of body mass index (BMI) with apnoea-hypopnoea index (AHI) and the Epworth sleepiness score, Table S1: Association of the Epworth sleepiness score, the Beck Depression Inventory-Fast Screen (BDI-FS) score, the State and Trait Anxiety Inventory (STAI) score, and the State-Trait Depression Inventory (IDER) score with the apnoea-hypopnoea index adjusted for sex (Model 1), adjusted for age (Model 2), adjusted for continuous positive airway pressure use (Model 3) and adjusted for body mass index (Model 4), Table S2: Association of changes in the Epworth sleepiness score, the Beck Depression Inventory-FS (BDI-FS) score, the State and Trait Anxiety Inventory (STAI) score, and the State-Trait Depression Inventory (IDER) score with changes in the apnoea-hypopnoea index after a post-continuous positive airway pressure therapy adjusted for sex (Model 1), adjusted for age (Model 2), adjusted for continuous positive airway pressure use (Model 3) and adjusted for body mass index (Model 4).
