# Supplementary material for: Anxiety and Depression in Patients with Obstructive Sleep Apnoea before and after Continuous Positive Airway Pressure: The ADIPOSA Study
Source: J Clin Med. 2019 Dec 1;8(12):2099. doi: 10.3390/jcm8122099 (PMC6947599; doi:10.3390/jcm8122099)
Supplement: Supplementary file 1 [file jcm-08-02099-s001.pdf]

Supplementary Table S1. Association of the Epworth sleepiness score, the Beck Depression Inventory-Fast Screen (BDI-FS) score, the State and Trait Anxiety Inventory (STAI) score, and the State-Trait Depression Inventory (IDER) score with the apnoea-hypopnoea index adjusted for sex (Model 1), adjusted for age (Model 2), adjusted for continuous positive airway pressure use (Model 3) and adjusted for body mass index (Model 4). P from multiple regression analyses.

	Apnoea-hypopnoea index			
	Model 1	Model 2	Model 3	Model 4
Epworth sleepiness score	0.894	0.874	0.988	0.749
BDI-FS score	0.785	0.835	0.880	0.741
STAI-State score	0.504	0.756	0.743	0.874
STAI-Trait score	0.171	0.231	0.227	0.229
IDER-State score	0.503	0.616	0.670	0.366
IDER-Trait score	0.957	0.900	0.950	0.915

Supplementary Table S2. Association of changes in the Epworth sleepiness score, the Beck Depression Inventory-FS (BDI-FS) score , the State and Trait Anxiety Inventory (STAI) score, and the State-Trait Depression Inventory (IDER) score with changes in the apnoea-hypopnoea index after a post-continuous positive airway pressure therapy adjusted for sex (Model 1), adjusted for age (Model 2), adjusted for continuous positive airway pressure use (Model 3) and adjusted for body mass index (Model 4). P from a multiple regression analysis.

	Δ Apnoea-hypopnoea index			
	Model 1	Model 2	Model 3	Model 4
Δ Epworth sleepiness score	0.630	0.535	0.663	0.610
Δ BDI-FS score	0.978	0.968	0.983	0.935
Δ STAI-State score	0.291	0.251	0.299	0.398
Δ STAI-Trait score	0.310	0.571	0.740	0.164
Δ IDER-State score	0.516	0.510	0.607	0.370
Δ IDER-Trait score	0.782	0.897	0.555	0.927

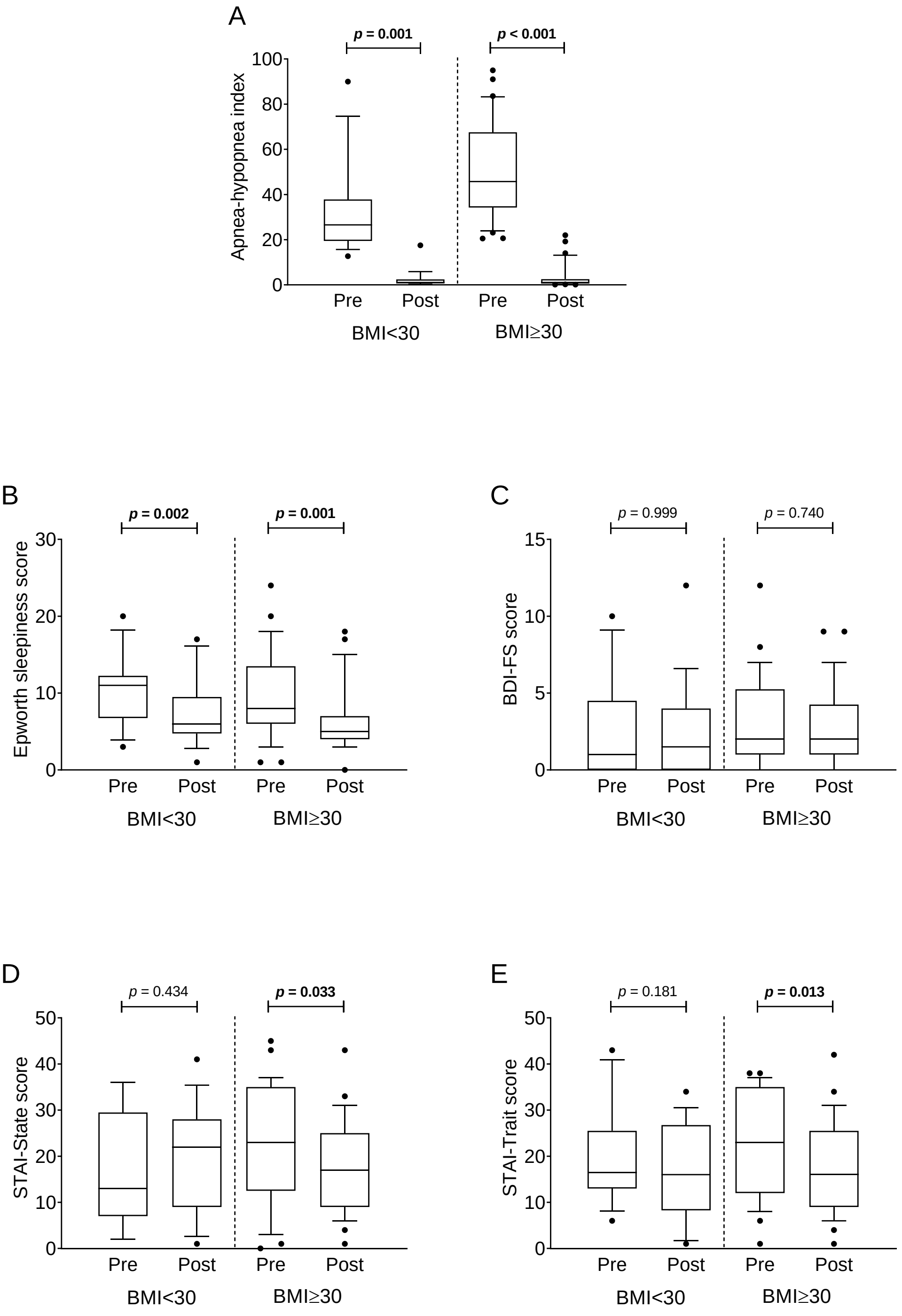

Supplementary Figure S1. Apnoea-hypopnoea index (Panel A), Epworth sleepiness score (Panel B), Beck Depression Inventory-FS (BDI-FS) score (Panel C), and State and Trait Anxiety Inventory (STAI) scores (Panels D and E) before and after the continuous positive airway pressure therapy comparing patients with obstructive sleep apnoea having a body mass index (BMI) <30 kg/m2 vs. ≥30 kg/m2. P value of Student's paired t-test. The data are shown as median ± interquartile range.

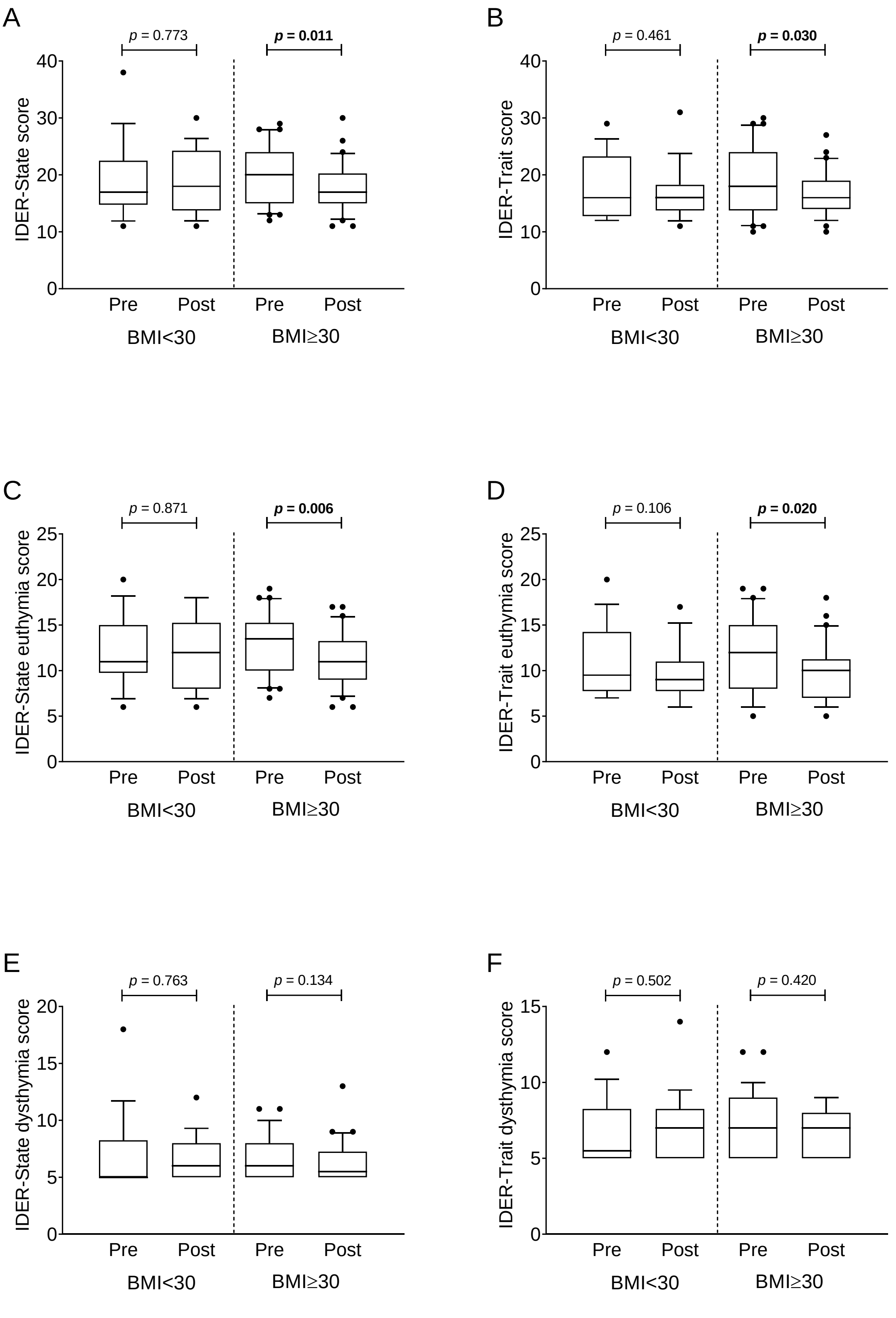

Supplementary Figure S2. State-Trait Depression Inventory (IDER) scores (Panels A and B), including euthymia and dysthymia specific values (Panels C, D, E and F) before and after the continuous positive airway pressure therapy comparing patients with obstructive sleep apnoea having a body mass index (BMI) <30 kg/m2 vs. ≥30 kg/m2. P value of Student's paired t-test. The data are shown as median ± interquartile range.

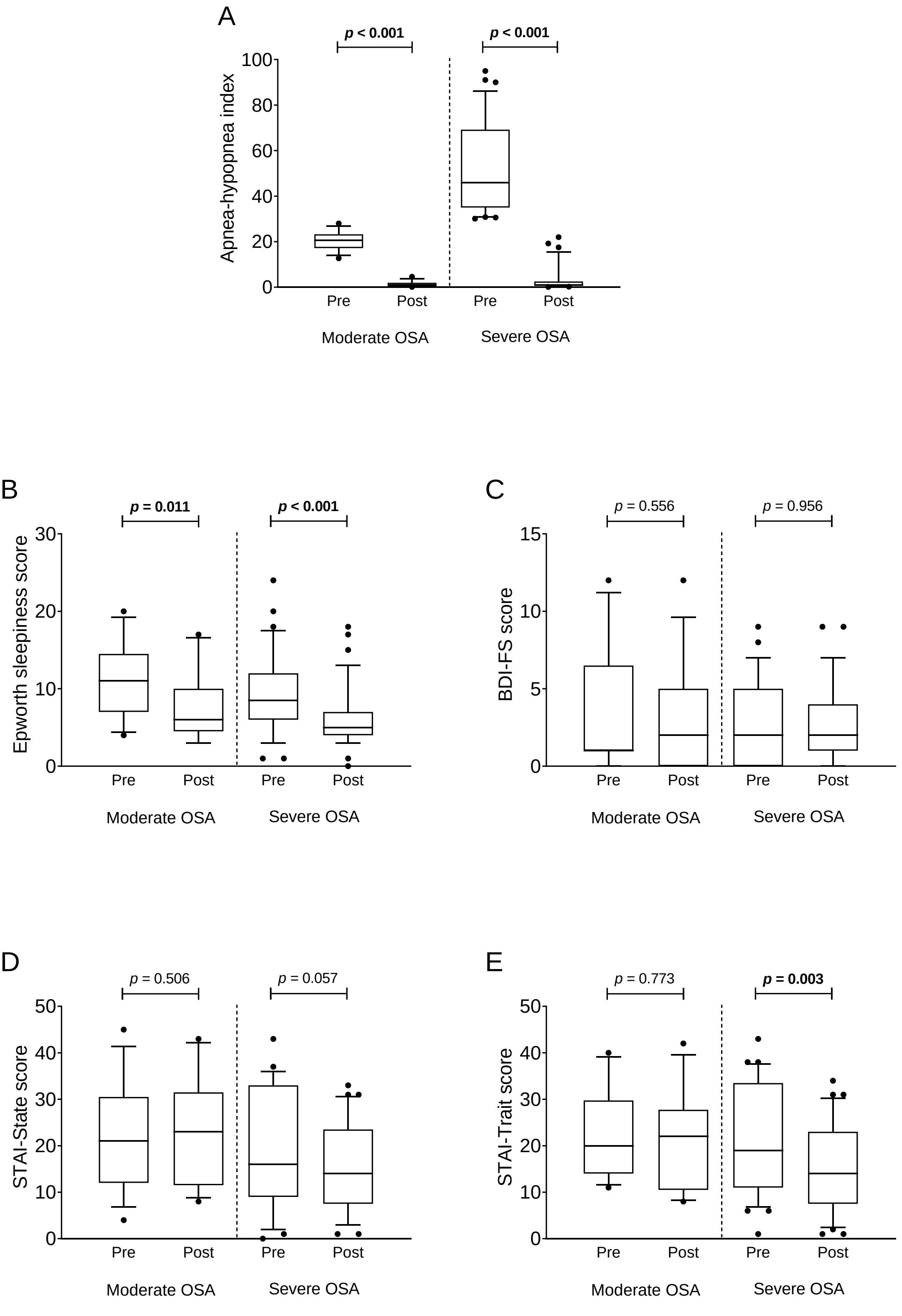

Supplementary Figure S3. Apnoea-hypopnoea index (Panel A), Epworth sleepiness score (Panel B), Beck Depression Inventory-FS (BDI-FS) score (Panel C), and State and Trait Anxiety Inventory (STAI) scores (Panels D and E) before and after the continuous positive airway pressure therapy comparing patients with moderate (AHI<30) vs. severe (AHI≥30) obstructive sleep apnoea (OSA). P value of Student's paired t-test. The data are shown as median ± interquartile range.

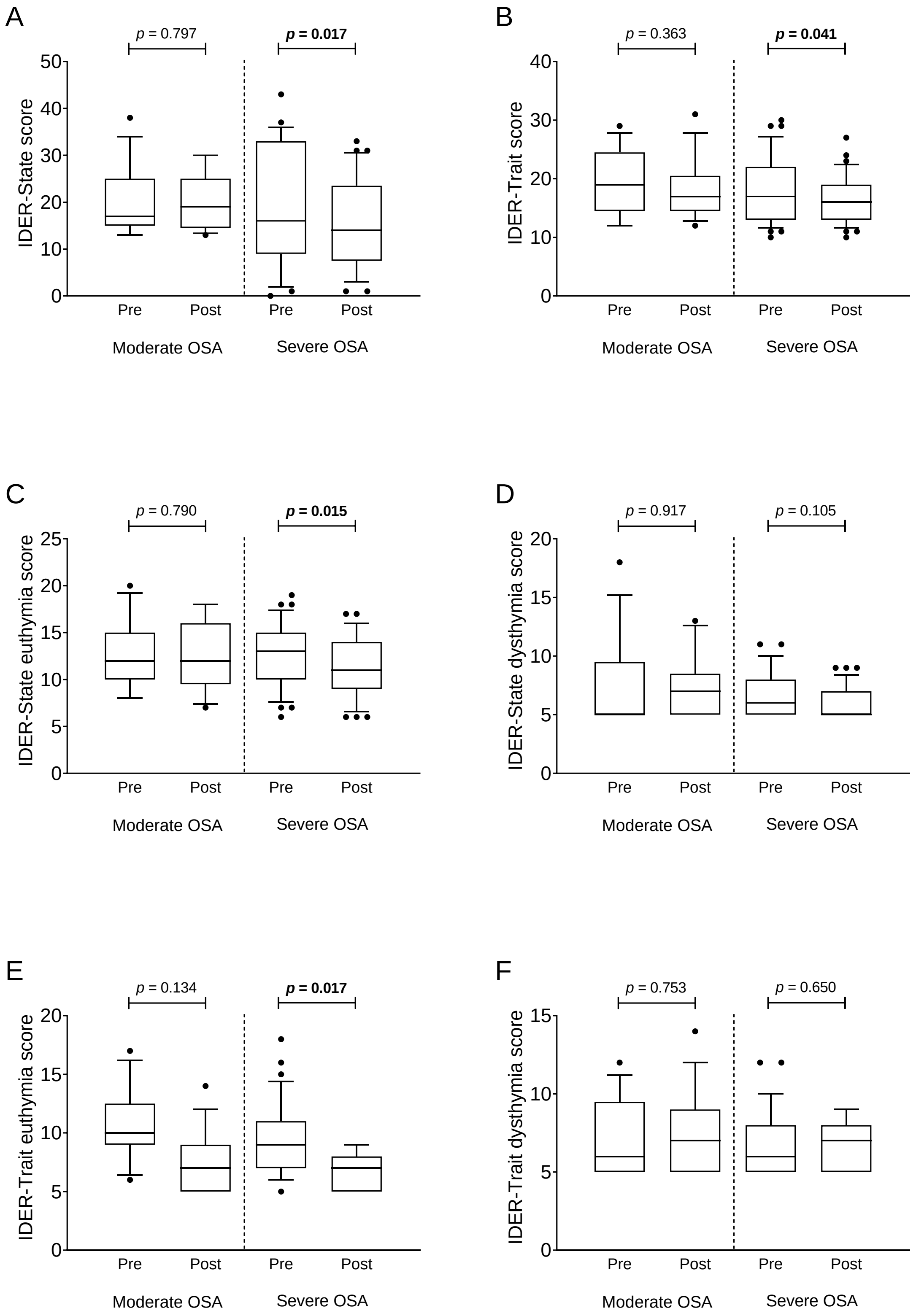

Supplementary Figure S4. State-Trait Depression Inventory (IDER) scores (Panels A and B), including euthymia and dysthymia specific values (Panels C, D, E and F) before and after the continuous positive airway pressure therapy comparing patients with moderate (AHI<30) vs. severe (AHI≥30) obstructive sleep apnoea (OSA). P value of Student's paired t-test. The data are shown as median ± interquartile range.

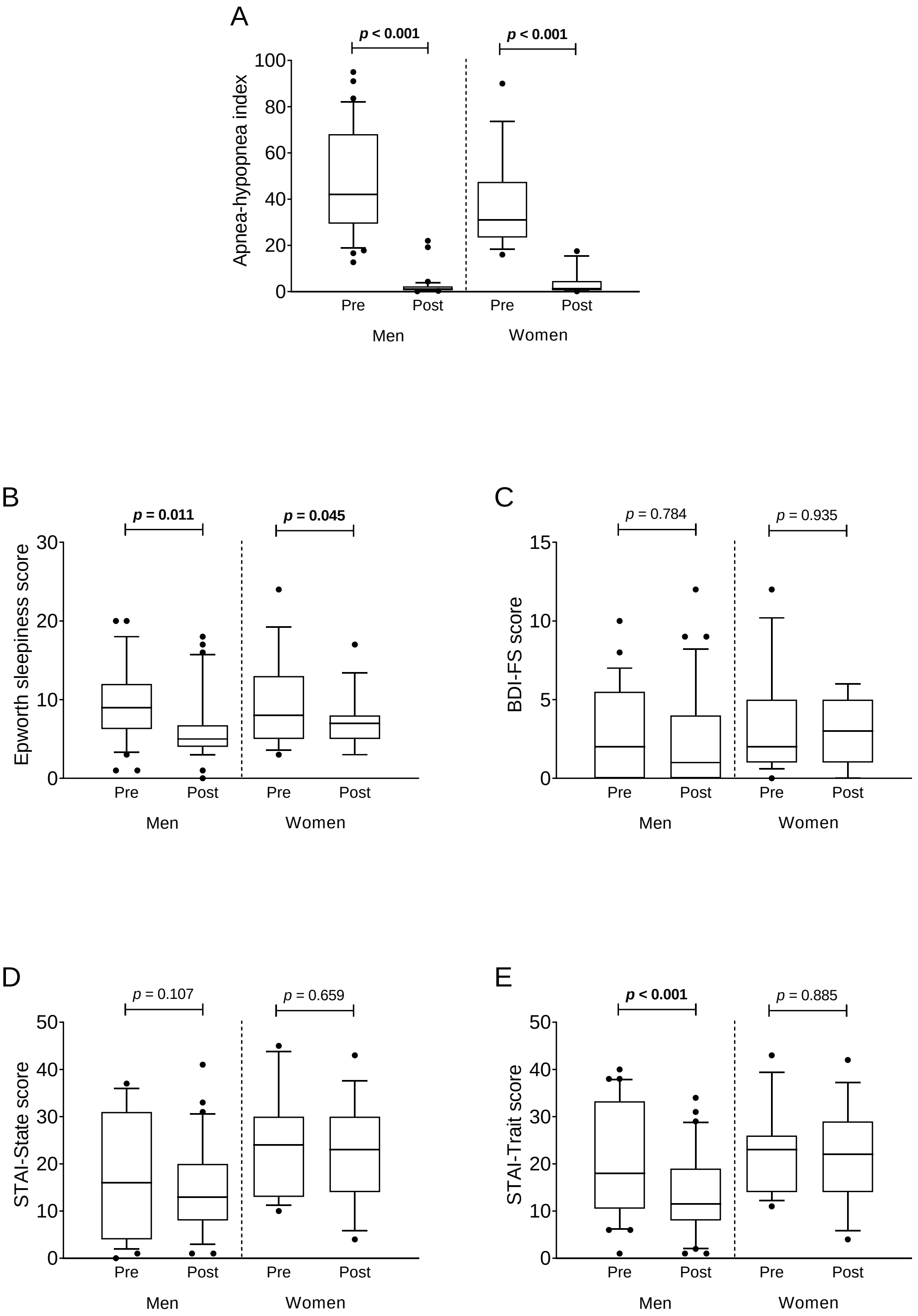

Supplementary Figure S5. Apnoea-hypopnoea index (Panel A), Epworth sleepiness score (Panel B), Beck Depression Inventory-FS (BDI-FS) score (Panel C), and State and Trait Anxiety Inventory (STAI) scores (Panels D and E) before and after the continuous positive airway pressure therapy men vs. women. P value of Student's paired t-test. The data are shown as median ± interquartile range.

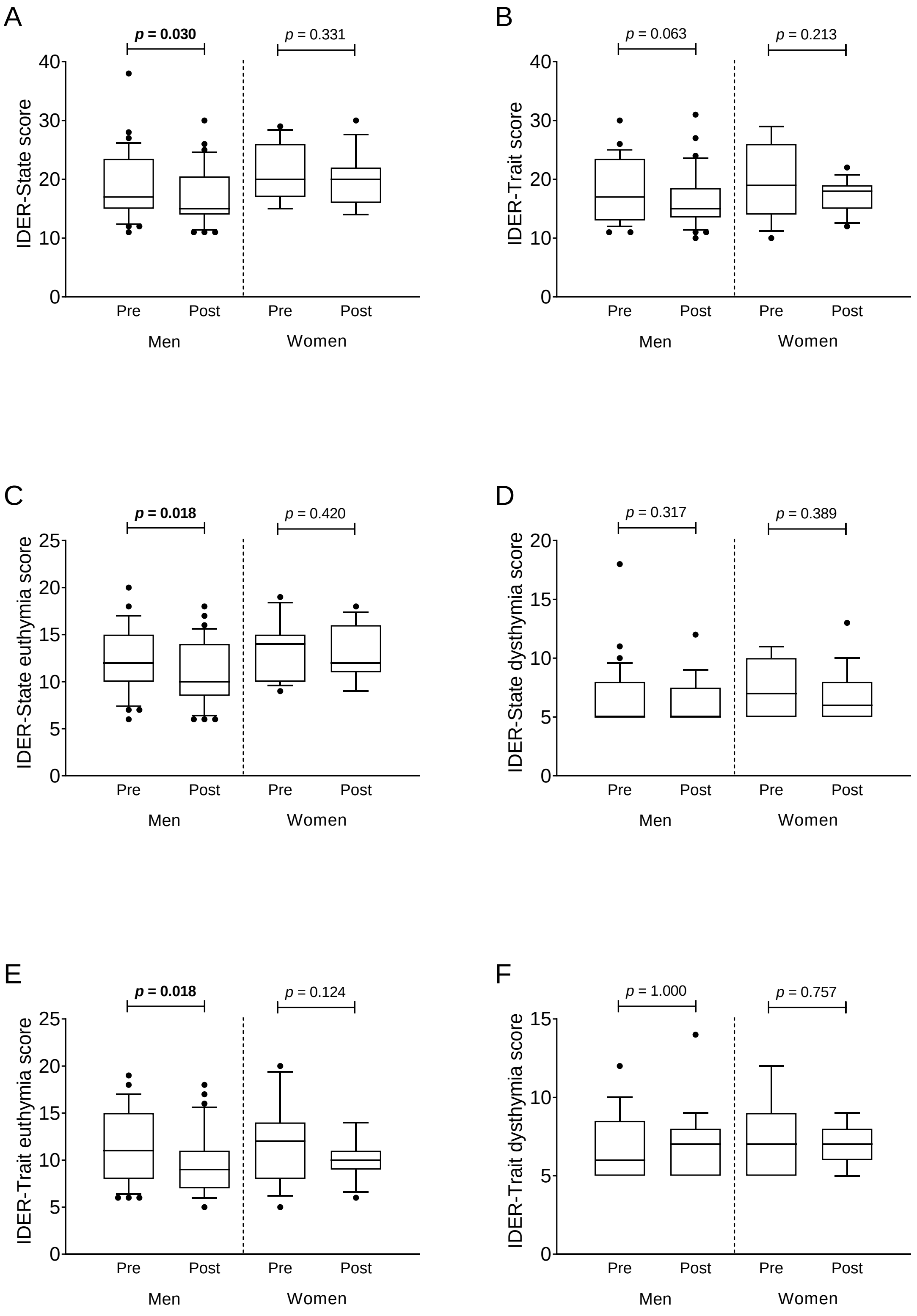

Supplementary Figure S6. State-Trait Depression Inventory (IDER) scores (Panels A and B), including euthymia and dysthymia specific values (Panels C, D, E and F) before and after the continuous positive airway pressure therapy men vs. women. P value of Student's paired t-test. The data are shown as median ± interquartile range.

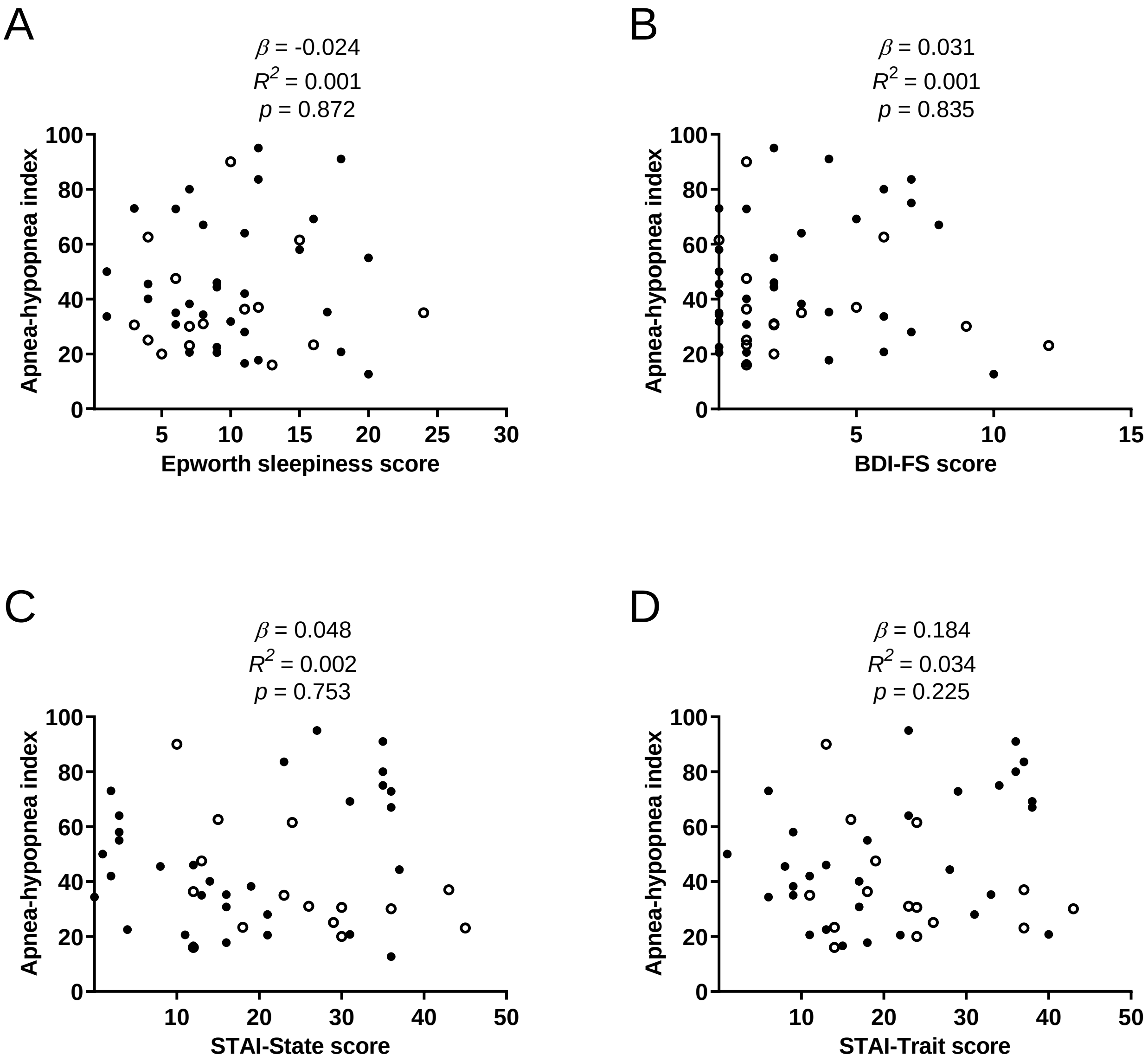

Supplementary Figure S7. Association of the Epworth sleepiness score (Panel A), the Beck Depression Inventory-FS (BDI-FS) score (Panel B), and the State and Trait Anxiety Inventory (STAI) scores (Panels C and D) with the apnoea-hypopnoea index. β (standardized regression coefficient), R2, and P from a simple linear regression analysis. Closed and open circles represent men and women, respectively.

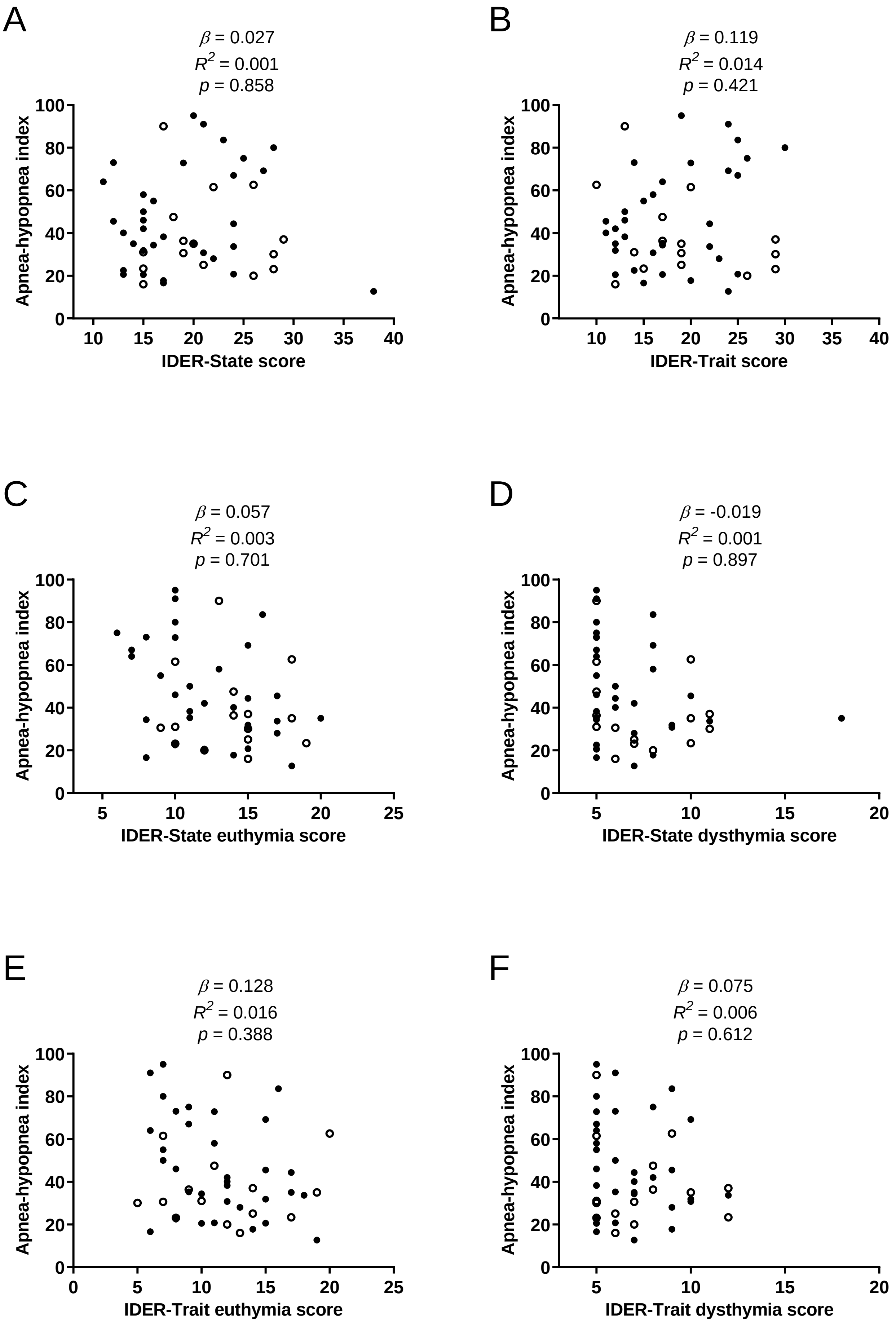

Supplementary Figure S8. Association of the State-Trait Depression Inventory (IDER) scores (Panels A and B) including euthymia and dysthymia specific values (Panels C, D, E and F) with the apnoea-hypopnoea index. β (standardized regression coefficient), R2, and P from a simple linear regression analysis. Closed and open circles represent men and women, respectively.

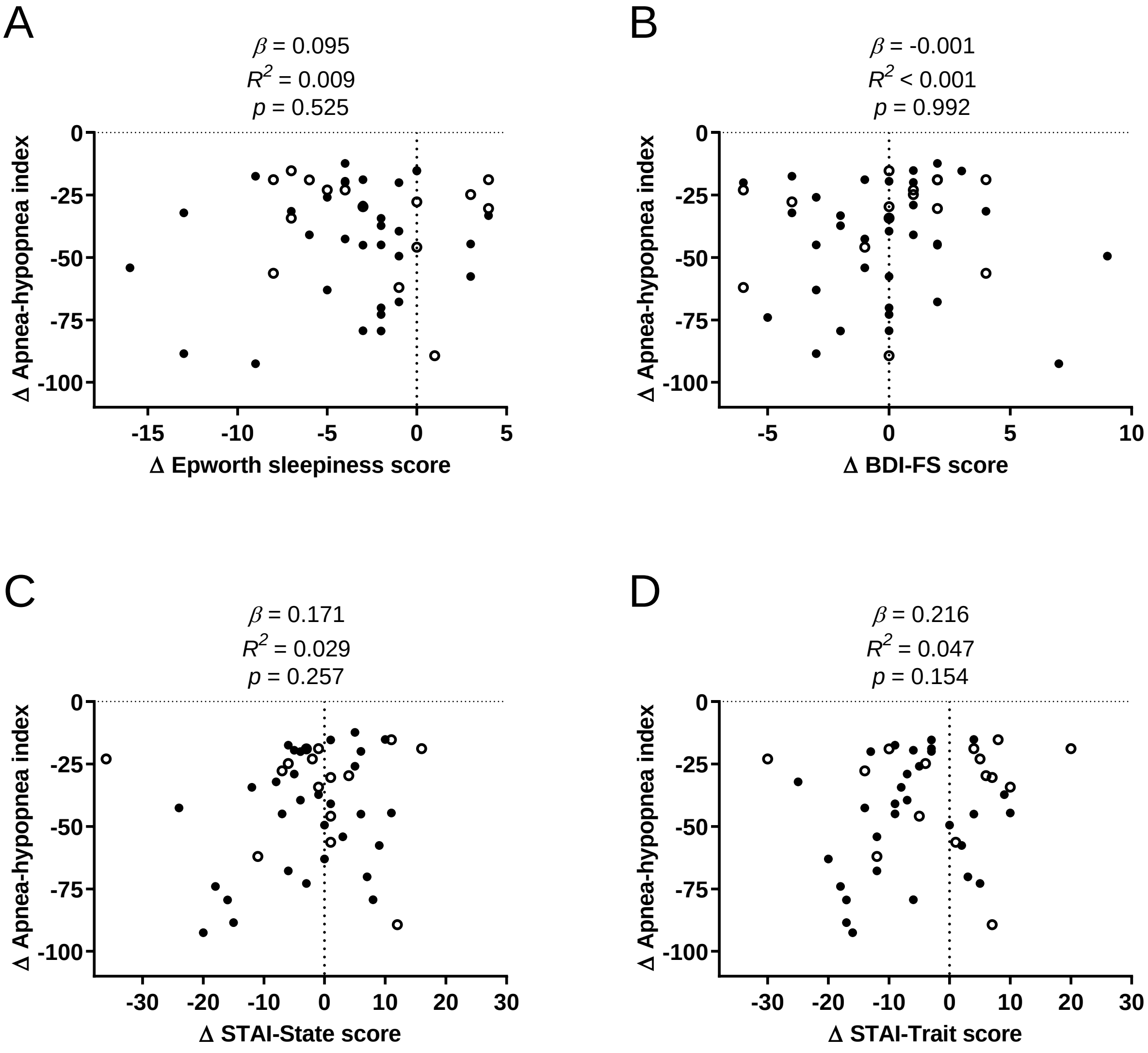

Supplementary Figure S9. Association of changes in the Epworth sleepiness score (Panel A), the Beck Depression Inventory-FS (BDI-FS) score (Panel B), and the State and Trait Anxiety Inventory (STAI) scores (Panels C and D) with changes in the apnoea-hypopnoea index after a post-continuous positive airway pressure therapy. β (standardized regression coefficient), R2, and P from a simple linear regression analysis. Closed and open circles represent men and women, respectively.

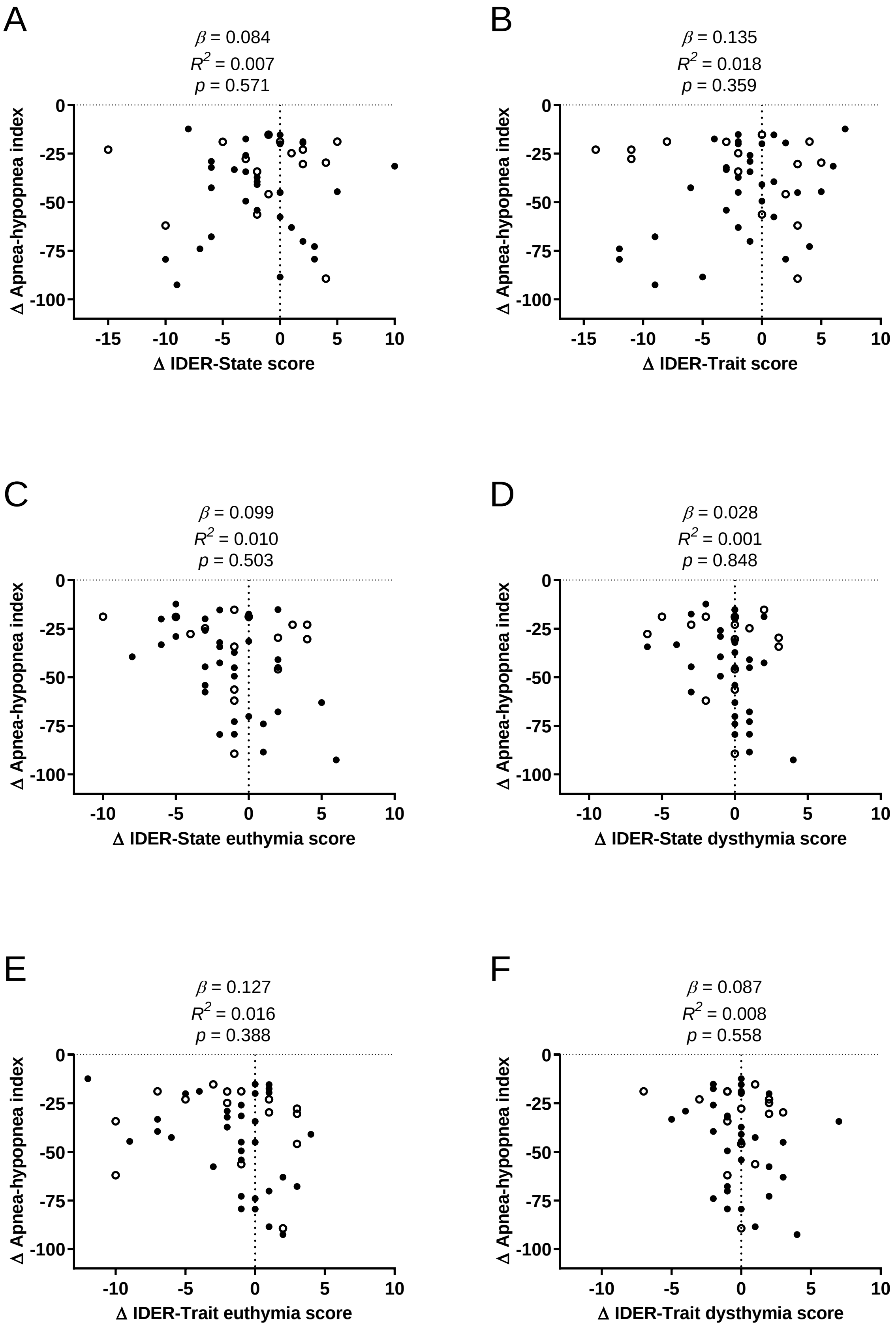

Supplementary Figure S10. Association of changes in the State-Trait Depression Inventory (IDER) scores (Panels A and B) including euthymia and dysthymia specific values (Panels C, D, E and F) with changes in the apnoea-hypopnoea index after a post-continuous positive airway pressure therapy. β (standardized regression coefficient), R2, and P from a simple linear regression analysis. Closed and open circles represent men and women, respectively.

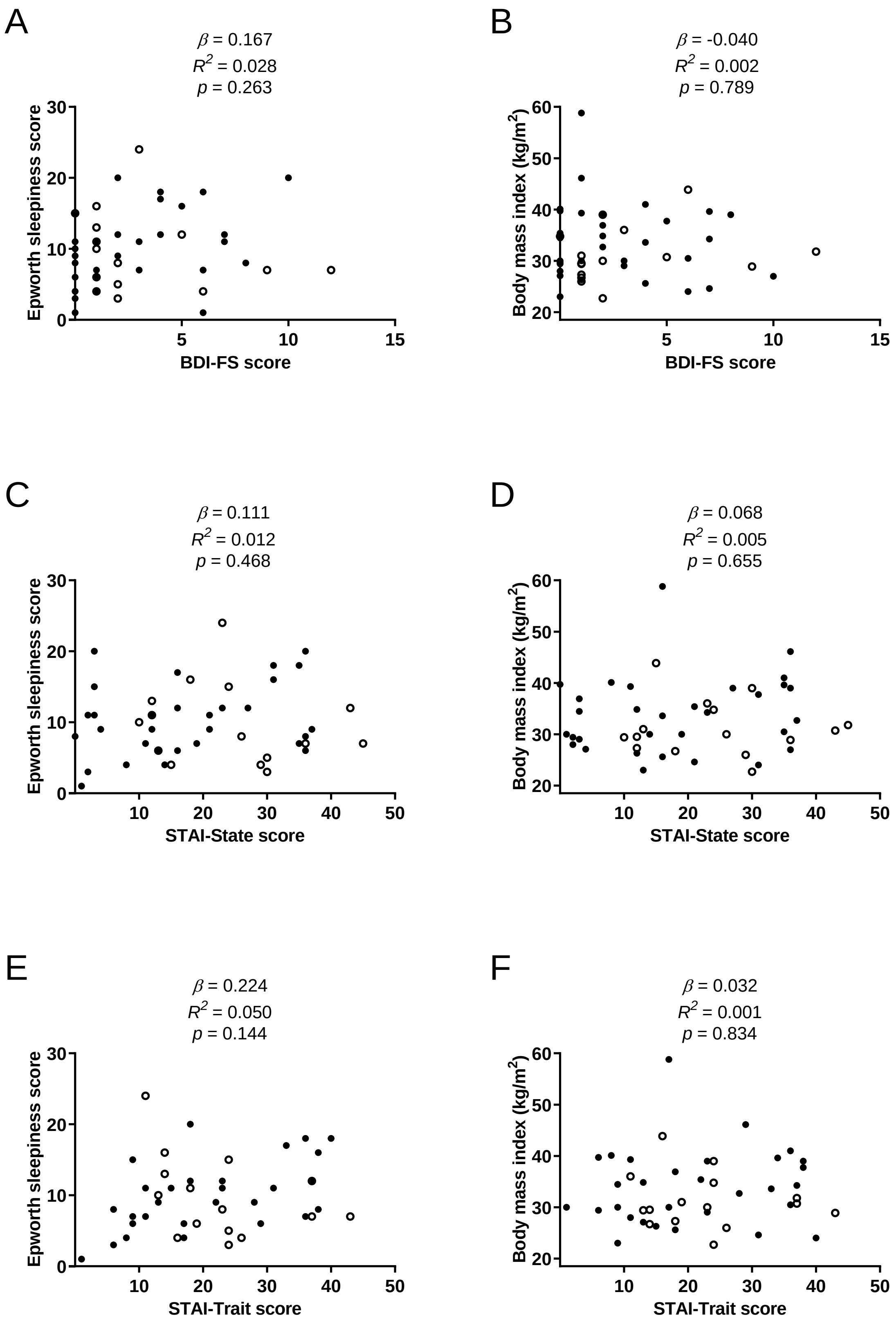

Supplementary Figure S11. Association of the Beck Depression Inventory-FS (BDI-FS) score (Panel A and B), and the State and Trait Anxiety Inventory (STAI) scores (Panels C, D, E and F) with the Epworth sleepiness score and the body mass index. β (standardized regression coefficient), R2, and P from a simple linear regression analysis. Closed and open circles represent men and women, respectively.

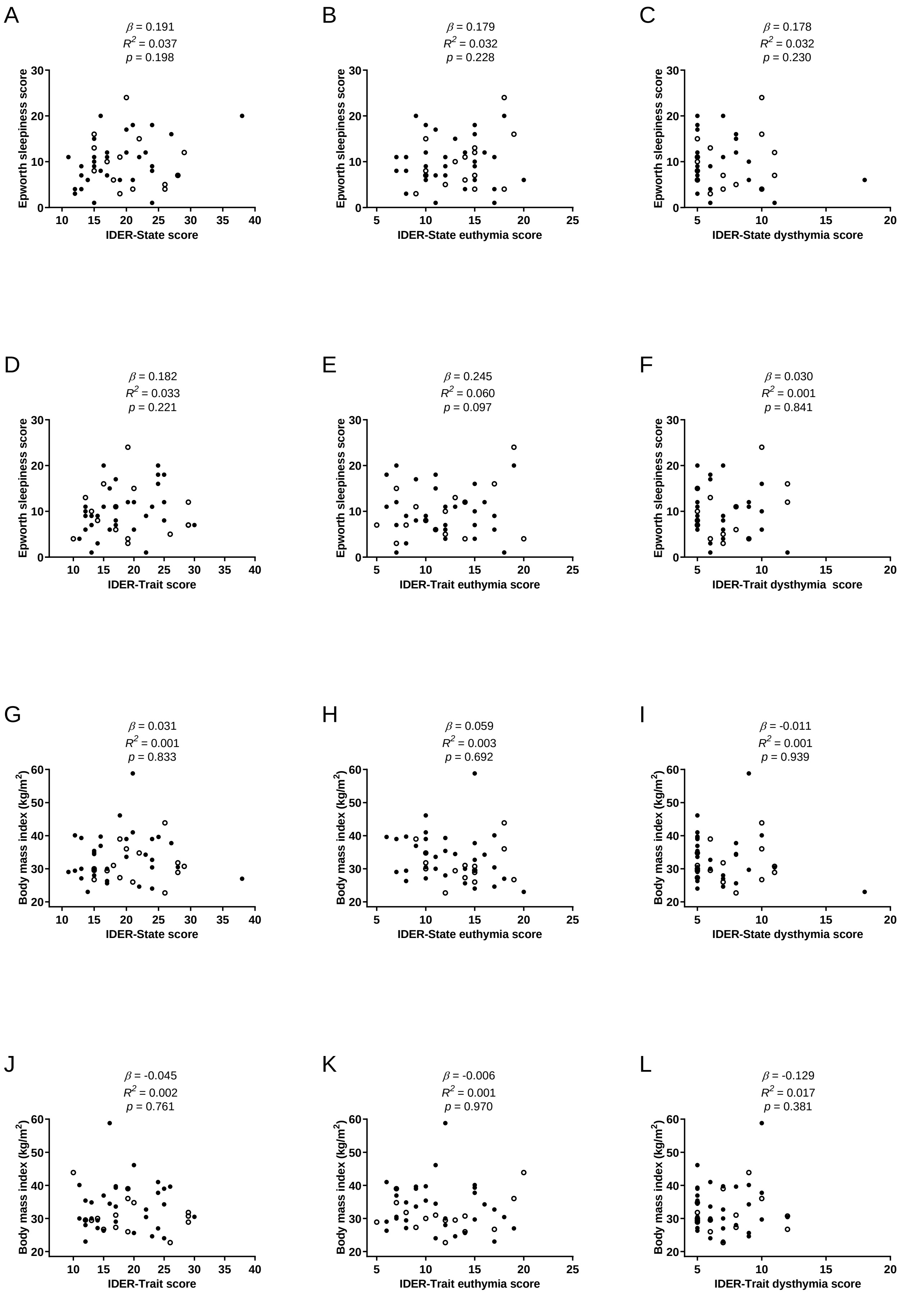

Supplementary Figure S12. Association of the State-Trait Depression Inventory (IDER) scores including euthymia and dysthymia specific values with the Epworth sleepiness score and the body mass index. β (standardized regression coefficient), R2, and P from a simple linear regression analysis. Closed and open circles represent men and women, respectively.

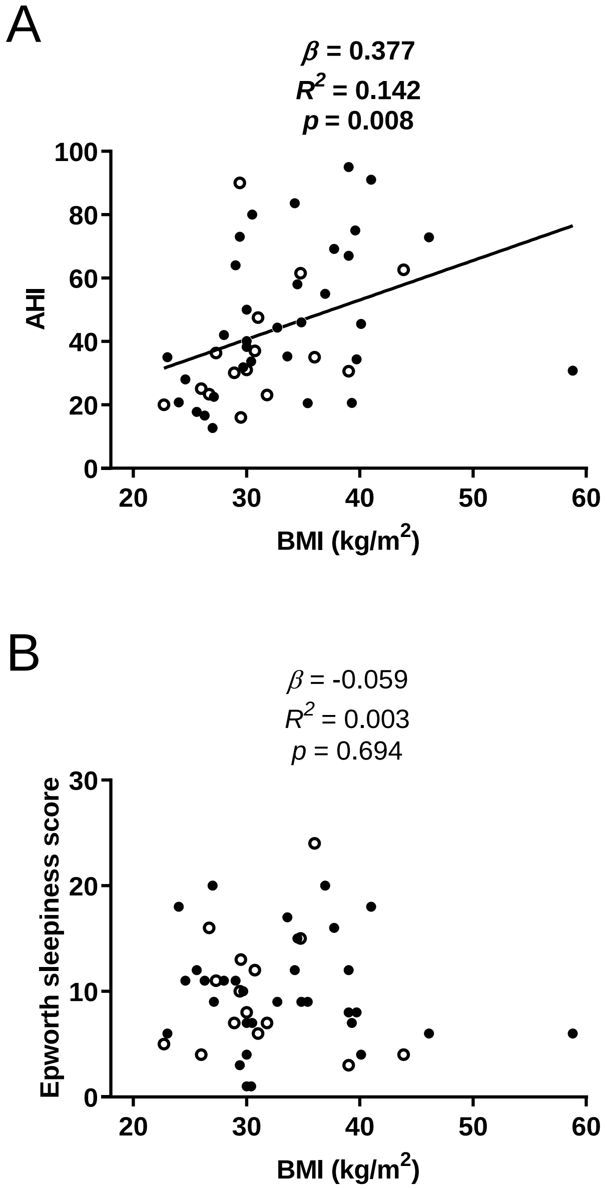

Supplementary Figure S13. Association of body mass index (BMI) with apnoea-hypopnoea index (AHI) (Panel A) and the Epworth sleepiness score (Panel B). β (standardized regression coefficient), R2, and P from a simple linear regression analysis. Closed and open circles represent men and women, respectively.
